# *E. coli* Cell Lysis Induced by Lys394 Enzyme Assisted by Magnetic Nanoparticles Exposed to Non-Heating Low-Frequency Magnetic Field

**DOI:** 10.3390/pharmaceutics15071871

**Published:** 2023-07-03

**Authors:** Azizbek D. Usvaliev, Natalia G. Belogurova, Konstantin V. Pokholok, Alexander V. Finko, Andrey N. Prusov, Dmitry Yu. Golovin, Konstantin A. Miroshnikov, Yuri I. Golovin, Natalia L. Klyachko

**Affiliations:** 1 School of Chemistry, Lomonosov Moscow State University, Moscow 119991, Russia; azximik@gmail.com (A.D.U.); nbelog@mail.ru (N.G.B.); kpokholok@gmail.com (K.V.P.); finko.alexander@gmail.com (A.V.F.); yugolovin@yandex.ru (Y.I.G.); 2 Skolkovo Institute of Science and Technology, Moscow 121205, Russia; 3 A.N. Belozersky Institute of Physico-Chemical Biology, Lomonosov Moscow State University, Moscow 119992, Russia; prusov@genebee.msu.su; 4 Institute of Nanomaterials and Nanotechnologies, G.R. Derzhavin Tambov State University, Tambov 392000, Russia; tarlin@yandex.ru; 5 Shemyakin and Ovchinnikov Institute of Bioorganic Chemistry, Russian Academy of Sciences, Moscow 117997, Russia; kmi@ibch.ru

**Keywords:** rod-like magnetic nanoparticles, S-394 bacteriophage endolysin, non-heating low-frequency magnetic field, enzymatic lysis of Gram-negative bacteria, *E. coli* cell wall

## Abstract

The spreading of microbial pathogens with more and more resistance to traditional low-molecular antibiotic agents demands new approaches to antibacterial therapy. The employment of bacteriophage enzymes capable of breaking bacterial cell walls has attracted much interest within this context. The specific features of the morphology of Gram-negative bacteria prevent the effective direct usage of lytic enzymes and require assistance from additional helpers to facilitate cell lysis. The current work is devoted to the study of boosting the lysis of *Escherichia coli* (*E. coli*) JM 109 and MH 1 strains induced by Lys394 bacteriophage endolysin by means of rod-like (56 × 13 nm) magnetic nanoparticles (MNPs) activated by a non-heating low-frequency magnetic field (LF MF) with a frequency of 50 Hz and a flux density of 68.5 mT in a pulse–pause mode (1 s on and 0.3 s off). According to theoretical assumptions, the mechanism of MNP assistance is presumably based upon the disordering of the outer membrane that facilitates enzyme permeation into peptidoglycans to its substrate. It is found that the effect of the LF MF reaches an almost a twofold acceleration of the enzyme reaction, resulting in almost 80 and 70%, respectively, of lysed *E. coli* JM 109 and MH 1 cells in 21 min. An increase in the membrane permeability was proven by two independent experiments employing β-lactamase periplasmic enzyme leakage and Nile Red (NR) hydrophobic dye fluorescence. It is shown that the outer membrane disordering of *E. coli* caused by exposure to LF MF nanoparticle movement leads to almost complete (more than 80%) β-lactamase release out of the cells’ periplasm to the buffer suspension. Experiments with NR (displaying fluorescence in a non-polar medium only) reveal a drastic reduction in NR fluorescence intensity, reaching a change of an order of magnitude when exposed to LF MF. The data obtained provide evidence of changes in the bacterial cell wall structure. The result shown open up the prospects of non-heating LF MF application in enhancing enzyme activity against Gram-negative pathogens.

## 1. Introduction

The introduction of antibiotics into medical practice has saved many human lives from infectious diseases caused by pathogen microorganisms. However, bacteria develop resistance to antibiotics fast, and so to control the pathogenic bacteria population, there is a need to develop new types of antibacterial agents that bacteria cannot develop resistance to soon [1,2]. Metal or metal oxide nanoparticles [3,4], carbon nanotubes [5], short polypeptides [6], and bacterial topoisomerase inhibitors [7] are considered as a base for such agents that are alternatives to antibiotics. In recent years, porcine myeloid antimicrobial peptides have been mentioned with increasing frequency due to their strong antimicrobial activity [8].

The other potential alternative to antibiotics is bacteriophages and their endolysins. The latter are enzymes produced at the last stages of a bacteriophage’s life cycle to disintegrate bacterial cell walls through peptidoglycan hydrolysis [9]. The efficiency of endolysins in the inhibition of Gram-positive and Gram-negative bacteria proliferation is being currently studied on various model animals, and in recent years, some clinical trials of such enzymes have begun [10,11,12].

It is presumed that the parallel evolution of bacteriophages and their targets has resulted in the selection of extremely conservative and stable sites at peptidoglycans for bacteriophage enzymes to bind to [13]. Thus, the development of bacterial resistance to such enzymes is quite implausible, or bacteriophages would have disappeared as a result of natural selection. This hypothesis was proven by studies on *Streptococcus pneumoniae* and *Bacillus anthracis* cells subjected to low concentrations of endolysins. Even mutagenesis enhancement by means of methanesulfonic ester did not lead to variants resistant to endolysins, while the same technique induced an enhancement of the resistance of the studied bacteria to novobiocin and streptomycin by three to four orders of magnitude [9].

The enzyme-induced lysis of bacterial cells occurs in vivo at the specific moment of the last stage of a bacteriophage’s life cycle, and endolysins are transported from the cytoplasm to peptidoglycans through the inner membrane by employing specific biochemical systems. Unlike the above, the application of endolysins as antimicrobial agents is assumed to proceed from outside of the cell [14,15].

Phage therapy studies have advanced faster than expected; however, there are many aspects that still need to be elucidated. Theoretically, there is no bacterium species that cannot be lysed by at least some bacteriophages. In this respect, bacteriophages are much more efficient than antibiotics. Despite the wide variety of antibiotics that have been found, no one of them can suppress every bacterial pathogen. The most promising feature of bacteriophage therapy is its selectivity because bacteriophages can only kill the pathogen they can recognize [16,17].

Unlike Gram-positive bacteria, whose peptidoglycans are exposed to the environment, Gram-negative bacteria are additionally coated with an outer membrane composed of an asymmetric lipid bilayer. The outer face of this bilayer is formed by negatively charged lipopolysaccharides stabilized by bivalent cations that make an outer membrane of Gram-negative bacteria impermeable to most substances [18]. Only small hydrophilic molecules can cross the outer membrane on their own, but for any macromolecule, such as an enzyme, the outer membrane is an impenetrable barrier [19,20]. Hence, the effective usage of peptidoglycan hydrolases from the outside requires a preliminary breakage of the outer membrane integrity that is sufficient for enzyme molecules to permeate through it to the peptidoglycan layer. There are many agents capable of destabilizing the outer membrane, including polymyxins and their derivatives [21], cationic antimicrobial peptides [22], and chelating agents [23], as well as metal or metal oxide nanoparticles employed as nanocarriers of antimicrobial agents [24]. However, the above techniques for breaking the outer membrane lack specificity to pathogenic bacteria. Moreover, such agents can interact with the non-target cells of an organism.

The other fast-developing approach to affect membrane properties is the nanomagnetomechanical activation of magnetic nanoparticles (MNPs), including rod-like ones, by means of an external non-heating low-frequency magnetic field (LF MF) that causes rotational oscillations of the MNPs that interact with the membrane [25]. As follows from the theoretical model of MNPs’ interaction with biological membranes, such oscillations of MNPs associated with living cells or vesicles and exposed to LF MF with a frequency of f < 1 kHz can induce significant changes in the cell membrane structure. The latter can produce various biochemical responses including a manifold increase in membrane permeability, leading to morphological and functional changes in living cells [25,26]. The above effects are of great interest to targeted drug delivery and controlled release from nanocarriers, the remote control of therapeutic agent activity, cell-level therapy, the selective apoptosis of malignant cells, etc. [27,28,29,30].

It was discussed in [27] that anisometric particles have a number of advantages over spheroid ones. In particular, they are able to produce not only shear but also normal deformation within the membrane that could enhance their mechanical impact on the latter. According to the model approach, rod-like MNPs can be bound to the membrane by weak adhesion forces (Van der Waals, hydrogen bonds, or dispersion interactions). Usually, the surface modification of MNPs by some organic molecules is used for their stabilization. Such molecules carry a surplus charge that results in additional electrostatic forces between the MNPs and the membrane [31].

In this work, we studied the cell lysis of *E. coli* strains JM 109 [32] and MH 1 [33] induced by S-394 bacteriophage recombinant endolysin (Lys394 endolysin) and the outer membrane disordering caused by dopamine-functionalized MNPs activated by a non-heating low-frequency alternating magnetic field (LF MF).

## 2. Materials and Methods

In this work, LF MF with a frequency of 50 Hz and a flux density of 68.5 mT in a pulse–pause regime (1 s on and 0.3 s off) was used (Astra-50, Tambov, Russia). The temperature control of all the studied samples was carried out with a 0.1 °C accuracy. JM 109 and MH 1 strains of *E. coli* were used as a substrate for recombinant Lys394 endolysin. *E. coli* strains were kindly provided by Belogurov A.A. (National Cardiology Center, Moscow, Russia). All optical measurements were carried out using a SpectraMax M5 device produced by Molecular Devices (San Jose, CA, USA).

### 2.1. Protein Expression and Purification

Recombinant enzyme Lys394 was produced by the C41 (DE3) *E. coli* strain with the plasmid pET23 containing DNA from the S-394 bacteriophage genome. Producer cells were supplied by the molecular bioengineering laboratory of M.M. Shemyakin and the Yu.A. Ovchinnikov institute of Bioorganic Chemistry (Moscow, Russia). The cells were cultivated in 2 L conical flasks containing 0.5 L of LB-Lennox solution and ampicillin (100 µg/mL) at 37 °C following the procedure described in [34]. Briefly, the cell culture was allowed to proliferate under intensive aeration until it reached an optical density of 0.6–0.8 at 600 nm (OD600). Lys394 synthesis was induced by adding isopropyl β-D-1-thiogalactopyranoside at a final concentration of 0.8 mM (Merck, Darmstadt, Germany) after cultivation. The cells were then centrifuged at 3000× *g* for 15 min at 4 °C (Eppendorf (Hamburg, Germany)). The sediment was re-suspended in buffer solution containing 20 mM Tris/HCl (Merck, Darmstadt, Germany) and 200 mM NaCl (Merck, Darmstadt, Germany) at pH = 8.0 and processed by a 20 kHz ultrasound treatment (Techpan UD-20, Warsaw, Poland) in a pulse–pause regime (20 s and 20 s) to destroy the cells. Cell debris was separated by centrifugation at 7000× *g* for 20 s at 4 °C. The absence of the target protein in this debris was proven using electrophoresis in polyacrylamide gel in denaturing conditions. Supernatant was eluted using a 5 mL HisTrap HP chromatographic column (GE HealthCare, Chicago, IL, USA). Step elution of the target protein was carried out using from 20 to 200 mM imidazole (in Tris/HCl buffer containing 200 mM NaCl at pH 8.0). Fractions with lytic activity were consolidated, and imidazole was removed using a dialysis sac (Servapore MWCO 12–14 kDa, Oldenburg, Germany). Evaluation of the purity of the obtained product was carried out using electrophoresis in polyacrylamide gel in denaturing conditions (Mini-PROTEAN, BIO-RAD, Hercules, Clearwater, FL, USA). Coomassie Brilliant Blue G-250 (ThermoFisher, Waltham, MA, USA) was used for gel staining.

### 2.2. Cultivation of E. coli Cells as a Substrate for Lys394

A total of 20 mL of *E. coli* culture was added to 250 mL of LB-Lennox solution and held for 20 h at 37 °C. Cells were deposited using a Jouan BR4i (France) centrifuge for 15 min at 1500× *g* and re-suspended in 40 mL of 10 mM Tris/HCl buffer at pH 8.2. After repeating the above deposition–resuspension procedure, the cell culture was diluted using the same buffer to reach an optical density of OD600 = 1.0. The resulting suspension was divided into 200 µL aliquots and stored at −70 °C for a month. The suspension was then defrosted just before the measurements.

### 2.3. Endolysin Activity Assay

Lys394 endolysin activity was evaluated through the rate of the turbidity change in the *E. coli* suspension. The optimal conditions for the enzyme activity based on [34] were adjusted for our experiments. A total of 185 µL of 10 mM Tris/HCl buffer solution with pH = 8.2, 10 µL of cell suspension concentrate, and 5 µL of endolysin solution were placed into the wells of a Corning 3599 (Somerville, MA, USA) 96-well plate so that the resulting endolysin concentration varied from 0 to 60 µg/mL and the optical density was OD600 = 0.2–0.3. Upon thorough stirring at RT (room temperature), the rate of the enzymatic lysis of the bacterial cells was measured by recording the change in the suspension’s turbidity at 600 nm. In parallel, the spontaneous lysis of the bacterial cells without the enzyme was also measured. Enzyme activity was evaluated from the slope of the linear part of the time-dependence curve, as described in [35] in more detail. All experiments were repeated at least three times, and mean values are shown.

### 2.4. Synthesis and Characterization of Rod-Like MNPs Functionalized by Dopamine

MNP synthesis was conducted based on the methods described in [30,36,37] and adjusted for the current study. Briefly, 10 mL of 0.5 M FeCl_3_ solution was mixed with 10 mL of 0.04 M HCl in a three-neck flask, and 4 mg of dopamine hydrochloride was added to the solution. Then, the solution was diluted with 180 mL of deionized water at 80 °C and mixed for 2 h at 80 °C. Then, the solution was cooled to RT and its pH was adjusted to 7.4 by adding 1 M NaOH solution. The sediment formed was washed with deionized water (40 mL/portion), centrifuged for 5 min at 1000× *g*, and finally re-suspended in 40 mL of deionized water. A total of 2 mL of the resulting suspension was mixed with 50 µL of 0.04 M hydrazine hydrate (Merck, Darmstadt, Germany) and subjected to microwave irradiation in a Monowave 300 (AntonPaar, Graz, Austria) reactor. The sample was exposed to four cycles of microwave heating, providing a temperature increase up to 100 °C in 3–5 min, which was followed by gradual cooling to RT after 30 s at elevated temperature.

The saturation magnetization of the MNPs (*M*; emu/g) was determined using superconducting quantum interference device (SQUID) analysis, which was conducted using a Physical Property Measurement System (Quantum Design, San Diego, CA, USA) equipped with a magnetometric vibration device with a 2 nm amplitude of oscillation at 40 Hz at 300 K. Hysteresis loops of the dependence of the magnetization, *M*, upon the LF MF intensity, *H*, were measured in a range of up to 30 kOe.

The Mössbauer spectra of the MNPs were obtained using an Ms-1104 electrodynamic spectrometer (Rostov-na-Donu, Russia) with constant acceleration. To carry out the Mössbauer spectra measurements, the samples were tightly packed in special plexiglass mandrels with a diameter of 15 mm. The sample thickness was about 20 mg/cm^2^. ^57^Co (Ritverc, Saint Petersburg, Russia) in a rhodium matrix with an initial activity of 25 mCi was used as a source of gamma radiation. The Mössbauer spectra were processed using the UNIVEM-MS (Rostov–na–Donu, Russia) software. The following parameters were evaluated: isomer shift, *Is*; hyperfine magnetic field, *H*_h_; line width, *G*; and the relative area of the spectrum components, *S*. The approximation of the magnetic sextets was carried out using a ratio of the areas of the lines of S(1,6):S(2,5):S(3,4) = 3:2:1. Isomeric shifts are given relative to α-Fe at 300 K.

A JEOL JEM 1200 (JEOL, Akishima, Japan) transmission electron microscope (TEM) with a 100 keV electron energy was used for imaging the MNPs and examining their adsorption at the *E. coli* cell wall surface. The MNP concentration in the suspension applied to the TEM sampling grid was 10^8^ particles per mL. To enhance the appearance of *E. coli* cells in the image, a 2% uranyl acetate solution that scattered electrons efficiently [38] was added to the suspension. The ratio of the MNPs to the cell concentration was around 500. This suspension was applied to a copper TEM grid with a formvar coating and was mildly dried before imaging.

The hydrodynamic radius and concentration of the MNPs were assessed by means of nanoparticle tracking analysis (NTA) using a NanoSight NS500 device (Malvern Panalytical, Malvern, United Kingdom) with the following parameters: laser wavelength—532 nm, video duration—at least 60 s, camera shutter—1000, and gain—400. The data obtained were processed using the NTA Analytical Software package (version 2.3). Each sample was tested at least 3 times on renewed portions of the suspension.

### 2.5. Lys394-Induced Lysis of E. coli Cells in the Presence of MNPs

The effect of the modified MNPs on the catalytic activity of Lys394 was evaluated by comparing the rates of the enzymatic lysis of *E. coli* in the presence and absence of nanoparticles. For this, 186 µL of 10 mM Tris/HCl buffer (pH 8.2), 10 µL of *E. coli* concentrated suspension (up to OD600 0.25–0.26), 2 µL of MNP suspension (from 10^7^ to 10^15^ particles per mL), and 2 µL of 4.8 µg/mL Lys394 were added into the wells of a 96-well plate, and the kinetics of the turbidity change at 600 nm were monitored. The same reaction mixture without MNPs was prepared and measured as a reference.

### 2.6. Effect of LF MF on the Cell Lysis of E. coli

The effect of LF MF on the cell lysis of *E. coli* was tested by comparing two identical reaction mixtures with OD600 = 0.2–0.3 containing only a bacterial cell suspension in 10 mM Tris/HCl buffer (pH 8.2, 25 °C). One of the samples was exposed to LF MF for 20 min, while the other was used as a reference.

### 2.7. Effect of LF MF on the Cell Lysis of E. coli in the Presence of MNPs

The effect of LF MF on the cell lysis mediated by MNPs was studied by comparing the lysis rates in two identical mixtures composed of cells and MNPs with concentrations of 2 × 10^7^ cell/mL and 8 × 10^9^ particles/mL, respectively, in 10 mM Tris/HCl buffer (pH 8.2, 25 °C). One of the samples was exposed to LF MF for 20 min, while the other was used as a reference.

### 2.8. Effect of LF MF on the Cell Lysis of E. coli Induced by Lys394 in the Presence of MNPs

The effect of LF MF on the Lys394-induced cell lysis mediated by MNPs was studied by comparing the lysis rates in two identical mixtures prepared as described in Section 2.5. After Lys394 endolysin was added to the mixture, one of the samples was exposed to LF MF for 20 min, while the other was used as a reference. The suspension’s optical density was measured every 2 min during the LF MF exposure.

### 2.9. Study of the Outer Membrane Permeability of JM 109 E. coli Strain Exposed to LF MF

#### 2.9.1. Evaluation of the Release of β-Lactamase from *E. coli* Cell Periplasm

Changes in the permeability of the outer membrane of *E. coli* induced by MNPs exposed to LF MF were assessed by measuring the release of β-lactamase from the cell periplasm to the suspension medium. The samples prepared as described in Section 2.7 were exposed to LF MF for 5, 10, 15, and 20 min and then centrifuged at 10,000× *g* for 1 min to sediment the cells. The chromogenic substrate CENTA (2 µL with concentration of 2.5 µg/µL) (Merck, Darmstadt, Germany) was added to the supernatant (198 µL), and the change in the absorbance at 405 nm was measured [39,40]. A sample prepared and processed the same way except without exposure to LF MF was used as a reference.

#### 2.9.2. Nile Red Dye Fluorescence

The effect of MNPs on the outer membrane of *E. coli* when exposed to external LF MF was studied using Nile Red (NR) (ThermoFisher, Waltham, MA, USA) dye [41]. A total of 186 µL of Tris/HCl buffer, 10 µL of the *E. coli* cell suspension, 2 µL of MNP suspension with a final concentration of 8 × 10^9^ particles per mL, and 2 µL of 40 µg/mL NR dye (NR was taken from a stock solution with a concentration of 0.1 mg/mL in acetone) were added into the wells of a 96-well plate (96-well black plate with a clear flat bottom, CORNING, Somerville, MA, USA). The reaction mixture preincubated at RT for 5–7 min was exposed to LF MF for 9–10 min, and afterwards the NR fluorescence was measured at 612 nm using 545 nm as the λ of excitation.

A sample prepared and processed in the same way except without exposure to LF MF was used as a reference. In addition, the possible effect of each component of the reaction mixture on NR fluorescence was checked separately.

## 3. Results

### 3.1. Characterization of Magnetic Nanorods

**Figure 1:**
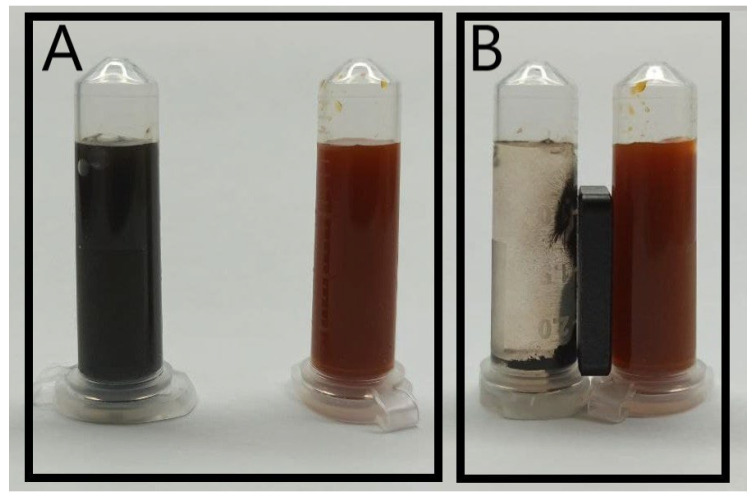
(**A**) Stable colloidal suspension of dopamine-coated particles at pH 7: β-akaganeite (orange) and final iron oxide (black) colloid. (**B**) The solutions with a neodymium magnet action.

The first stage of the synthesis resulted in a dark-orange suspension of non-magnetic nanoparticles (Figure 1B right) made of β-akaganeite. Black MNPs that were attracted to a steady magnet (Figure 1A,B left) were obtained at the second stage using microwave irradiation.

**Figure 2:**
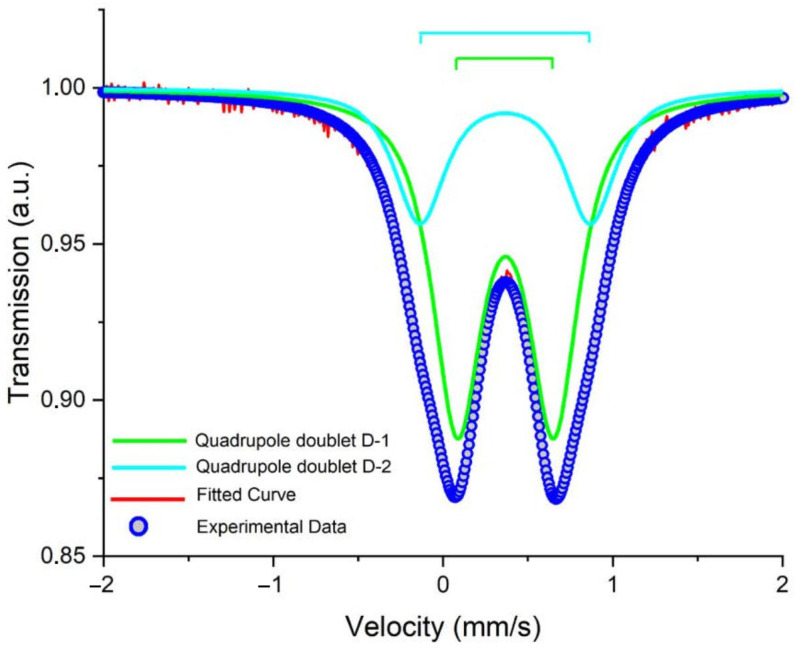
Experimental Mössbauer spectrum (referenced to α-Fe) of β-akaganeite at 300 K. The red solid line and the blue circle symbol scatter plot are the calculated and experimental spectra, respectively. The solid color lines show the total fit as well as the sub-spectral components.

The obtained nanoparticles were characterized using the Mössbauer spectroscopy method. Figure 2 presents the Mössbauer spectrum of the precursor obtained at 300 K. As can be seen, it was composed of a quadruple doublet with widened lines, the parameters of which are summed up in Table 1 (D-1, 2). The area of the D-1 component with lower quadruple splitting was around 68%, while that of D-2 was 32%. The chemical shifts of both the components were around 0.36–0.37 mm/s. These data were in good agreement with those reported in the literature [42,43] for β-akaganeite.

**Figure 3:**
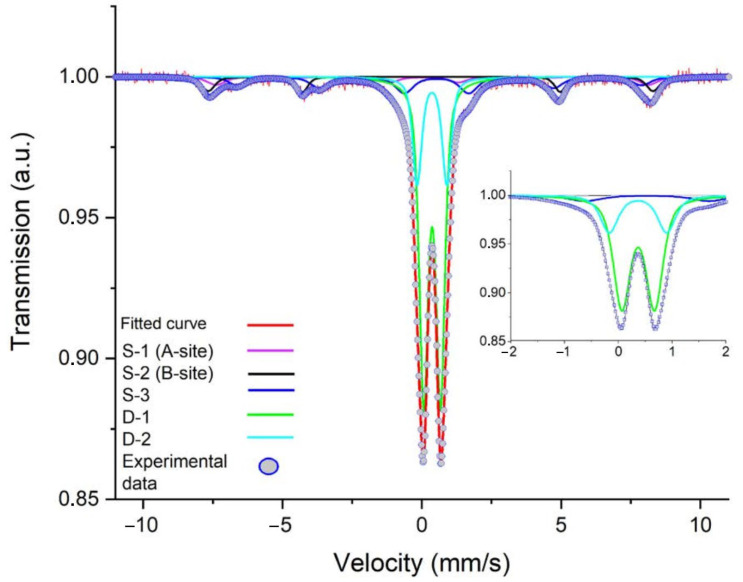
Mössbauer spectrum of nanorods recorded at 300 K, containing Fe^3+^ on octahedral B-sites (red) and tetrahedral A-sites (violet) in Fe_3_O_4_. The third component (S-3, blue) is a signal of Fe^3+^ in high-spin state, which was attributed to γ-Fe_2_O_3_. The solid lines D-1 and D-2 correspond to β-akaganeite. The circles represent the experimental data, whereas the solid lines are the calculated spectra. Insert demonstrates spectrum at more narrow velocity.

The complex multicomponent Mössbauer spectrum of the MNPs is presented in Figure 3. Table 2 sums up the data concerning the spectrum’s superfine structure. All isomeric shift-values are given with respect to metallic α-Fe at RT. This was approximated by the superpositioning of three sextets and two quadruple doublets. For the first sub-spectrum, the values of the isomeric shift and the hyperfine magnetic field corresponded to Fe^3+^ in the tetrahedral environment (A-site) of magnetite. This component had an intensity of 6%. The second component had an isomeric shift value of 0.58 mm/s and a hyperfine magnetic field value of 450 kOe, which are characteristic values for Fe^2.5+^ in an octahedral environment (B-site). This component had an intensity of 13%. The third sub-spectrum had an isomeric shift value of 0.33 mm/s and a hyperfine field value of 495 kOe with an intensity of 7%. The third component was attributed to maghemite. The ratio of sextets 1 and 2 was equal to two, which is close to the value for pure crystalline magnetite [44]. The hyperfine parameters of doublets 1 and 2 indicated that the precursor reacted partially, and its intensity in the MNPs was 74%. We can hence conclude that the studied sample consisted of three phases: 19% magnetite from the sum of the intensities of the A- and B-sites, 7% maghemite from the third component of the spectrum, and 74% β-akaganeite from the sum of the intensities of D-1 and D-2 [45,46].

**Figure 4:**
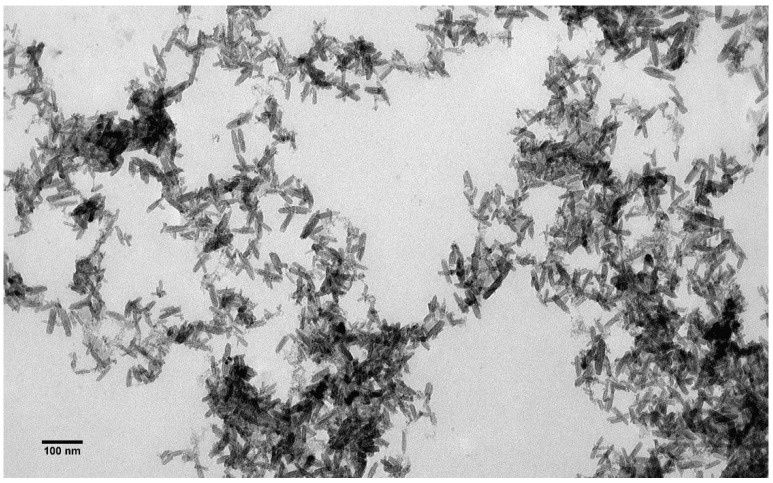
Transmission electron microscopy images of β-akaganeite nanoparticles.

**Figure 5:**
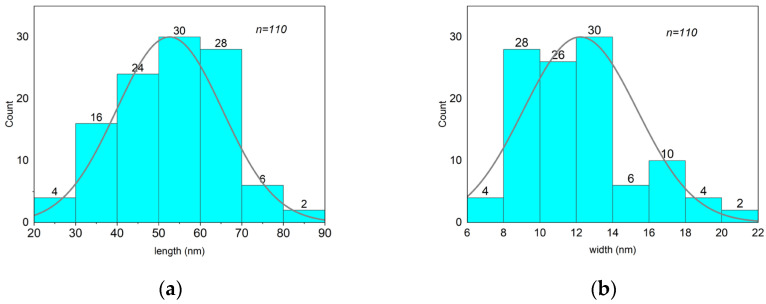
Analysis of the length and width distributions of β-akaganeite nanoparticles. The average size of nanoparticles was 53 ± 13 nm in length (**a**) and 13 ± 3 nm in width (**b**). The data for the histogram were obtained from a micrograph of β-akaganeite (Figure 4). The particle size was determined using ImageJ software. n—the number of particles.

The size and shape of the β-akaganeite nanoparticles were monitored by TEM. As can be seen in Figure 4, the β-akaganeite formed rod-like nanoparticles. Figure 5 presents the distributions of their length and width with average values of 53 ± 13 nm and 13 ± 3 nm, respectively. The hydrodynamic radius of the nanoparticles as determined by NTA was equal to 77 ± 8 nm, and the concentration was 6 × 10^15^ particles per mL.

**Figure 6:**
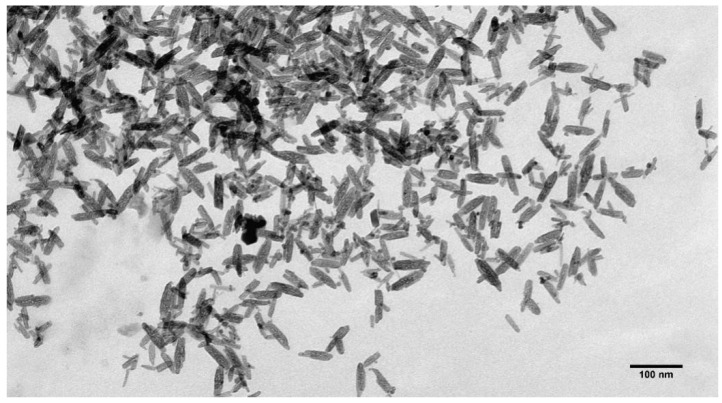
Transmission electron microscopy images of MNP nanorods.

**Figure 7:**
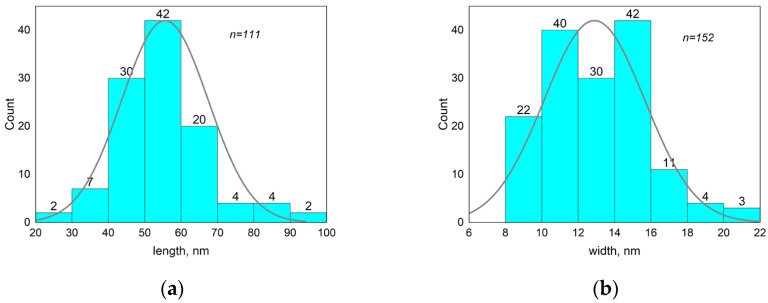
Analysis of the MNPs’ length and width distribution. The average size of nanoparticles was 56 ± 13 nm in length (**a**) and 13 ± 3 nm in width (**b**). The data for the histogram were obtained from the micrograph of the MNPs (Figure 6). Particle size was determined using ImageJ software.

The TEM images show that the rod-like particle shape was preserved in the MNPs (compare Figure 4 and Figure 6), and they had an average length of 56 ± 13 nm and width of 13 ± 3 nm (size distribution is shown in Figure 7). As can be seen (compare Figure 5 and Figure 7), the length distribution of the MNPs was narrower than that of the β-akaganeite nanoparticles. The MNPs with a length below 40 nm and above 60 nm were diminished significantly as a result of the partial reduction in the amount of β-akaganeite. Note, the average particle length grew slightly, while its width remained nearly the same for the MNPs and β-akaganeite, respectively. The measured average hydrodynamic radius of the MNPs was 84 ± 10 nm (77 ± 8 nm for β-akaganeite).

**Figure 8:**
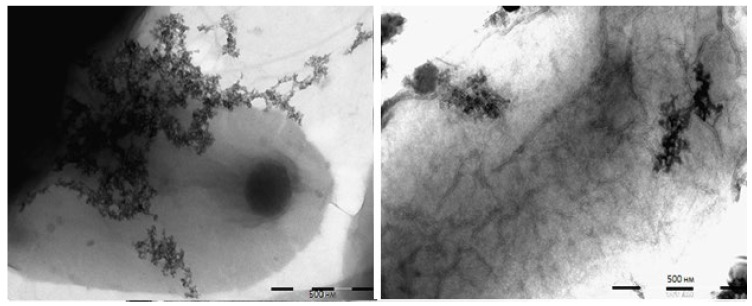
TEM images of interaction of MNPs with *E. coli* cell in the presence of 2% uranyl acetate as a contrast agent. The figures depict the same cell at different locations. The ratio of particles to cells was 500.

The MNPs mixed with the *E. coli* strain JM 109 was observed using TEM. Images of different parts of the same cell surface are shown in Figure 8. The MNPs can be seen together with the cell (interactions could be assumed).

**Figure 9:**
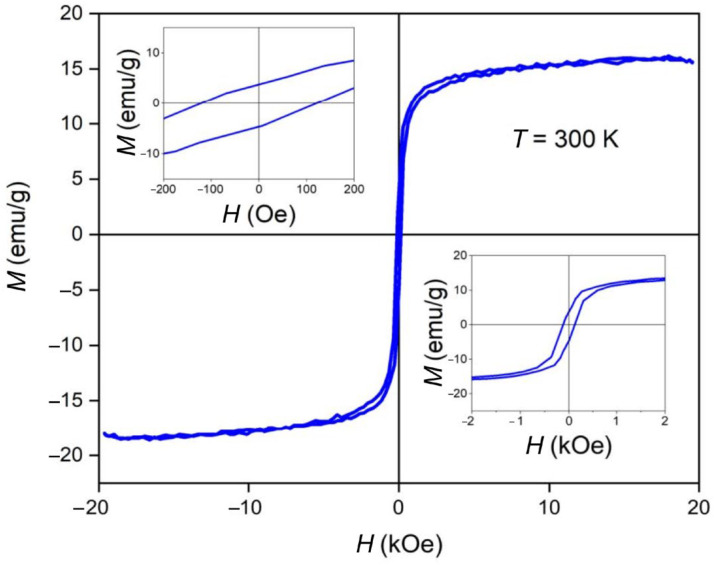
Magnetization versus field (*M–H*) plots of final nanorods at 300 K. Inserts demonstrate magnetization curve at more narrow field range.

The dependence of the magnetization of the MNPs upon the magnetic field intensity at 300 K is shown in Figure 9. The measured saturation magnetization was 15.6 emu/g. It should be noted that the above value was normalized to the total mass of the studied sample. Recalculation of this value on the mass of magnetic material gave a value of about 60 emu/g for the saturation magnetization, which was close to the value typical for a magnetite and maghemite mixture [47,48]. The coercive force (H_c_) of the sample was 120.9 Oe (9.6 kA/m). As can be seen (upper left and lower right inserts), the ferrimagnetic-type magnetic structure of the MNPs was proven.

### 3.2. Recombinant Endolysin Lys394 Isolation and Purification

**Figure 10:**
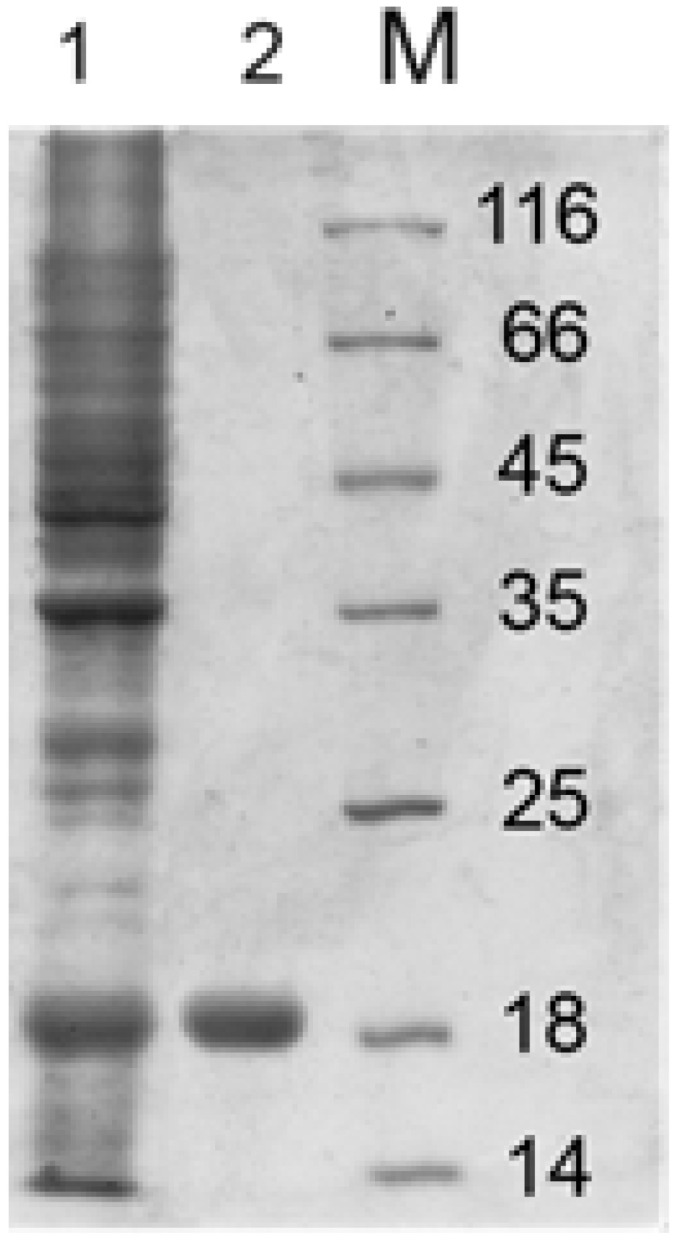
SDS-PAGE of Lys394 endolysin purification procedure: lane one—crude extract of *E. coli* C41(DE3) cells containing PEE3-Lys394 plasmid incubated with 0.5 mM IPTG for 3 h at 37 °C; lane two—the enzyme fraction after Ni^+2^-chelating chromatography; lane M—protein molecular mass standards.

Figure 10 presents the SDS-PAGE of Lys394 endolysin before and after purification. Lane one corresponds to the cell lysate. Lane two is the enzyme obtained after the affinity chromatography purification. As can be seen, the final product was a homogeneous specimen with a molecular mass around 18 kDa, which was in good agreement with [34].

### 3.3. Enzymatic Activity of Endolysin Lys394

**Figure 11:**
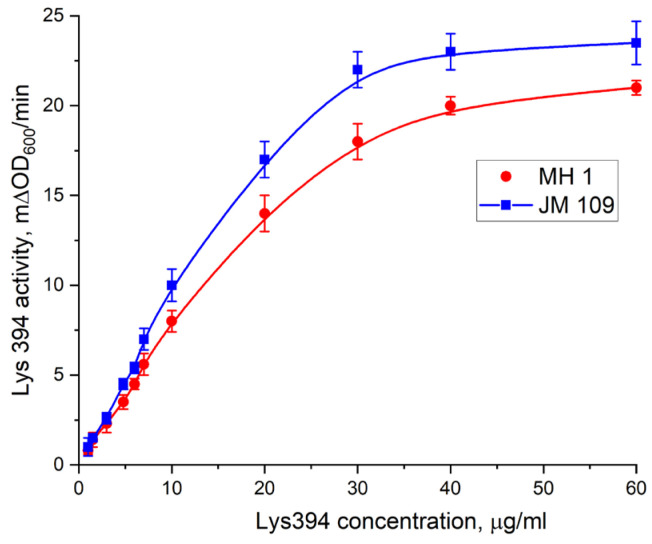
The dependence of the activity of Lys394 endolysin on the enzyme concentration in the reaction mixture on *E. coli* cells. Red spheres indicate MH 1, and blue squares indicate JM 109. Lysis was carried out in 10 mM Tris/HCl (pH 8.2) at 25 °C, and *E. coli* cell concentration was 2 × 10^7^ cells/mL. Each point on the graph corresponds to the average value of three measurements ± standard deviation.

The catalytic activity of the endolysin Lys394 on the enzyme concentration is shown in Figure 11 for the JM 109 and MH 1 *E. coli* cells. As can be seen, the lysis rates of both the JM 109 and MH 1 cells linearly depended on the enzyme concentration up to 20 µg/µL. Above that, the dependence tended towards saturation, reaching asymptotic values of 23.5 mΔOD600/min for JM 109 and 21 mΔOD600/min for MH 1, respectively. To study the effect of LF MF on the outer membrane permeability of *E. coli* in the presence of MNPs, the enzyme concentrations of 4.8 µg/µL and 6.0 µg/µL from the linear region of the dependence were chosen for JM 109 and MH 1, respectively. It should be noted that the enzyme we used contained six histidine residues that could be cut out by TEV-protease. As shown earlier, the latter did not affect the activity of Lys394 endolysin [34].

### 3.4. The Effect of MNPs on the Cell Lysis Induced by Lys394 Endolysin

**Figure 12:**
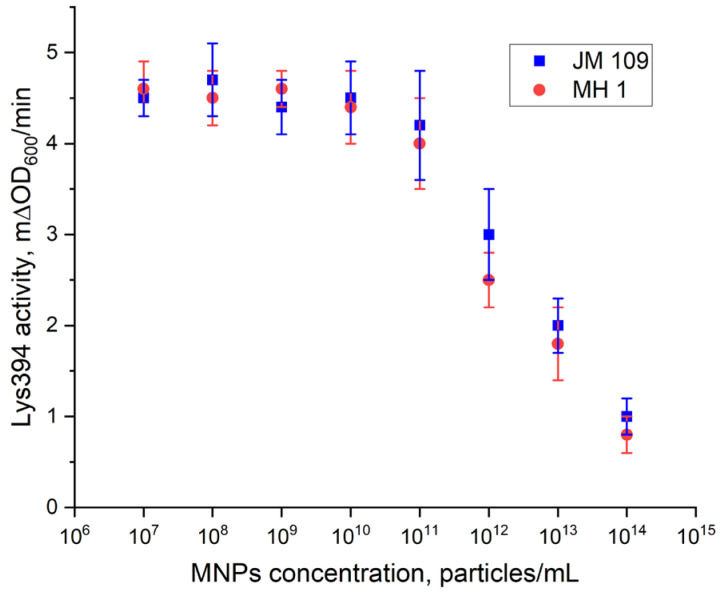
The dependence of the activity of Lys394 endolysin on the MNP concentration. Enzyme concentrations were 4.8 and 6.0 µg/mL for JM 109 and MH 1, respectively. The reaction was carried out in 10 mM Tris-HCl (pH 8.2) at 25 °C. The cell concentration was 2 × 10^7^ cells/mL.

Figure 12 presents the dependence of the lysis rate of the *E. coli* cells rate on the concentration of the MNPs. No effect of the MNPs on the cell lysis induced by Lys394 endolysin was found up to a concentration of MNPs of 10^11^ particles/mL. The lysis rate at these particle concentrations was equal to 4.5 mΔOD600/min, which was close to the rate obtained in the absence of MNPs at the same enzyme concentrations. A further increase in the MNP concentration resulted in a fast decrease in the lysis rate to 1.0 mΔOD600/min. The MNP concentration of 8 × 10^9^ particles per mL was chosen for the rest of the study.

### 3.5. Combined Effect of LF MF and MNPs on Spontaneous E. coli Cell Lysis

**Figure 13:**
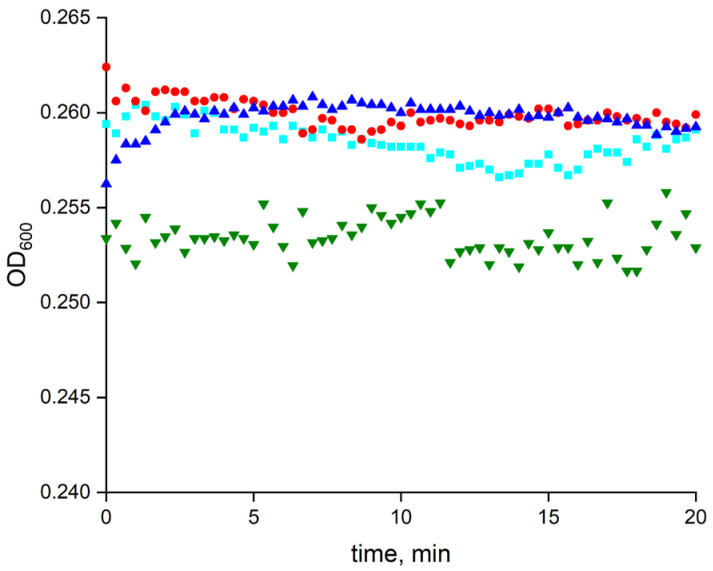
The effect of LF MF and MNPs on spontaneous lysis of *E. coli* cells. Red circles, cyan cubes, and green triangles correspond to spontaneous lysis of JM 109 cells in the presence of MNPs, LF MF, and combination of MNPs with LF MF, respectively. Blue triangles represent reference cells only without LF MF and MNPs. The reaction mixture was incubated in 10 mM Tris-HCl buffer (pH 8.2) at 25 °C. MNP and *E. coli* concentrations were 8 × 10^9^ particles/mL and 2 × 10^7^ cells/mL, respectively.

The combined effect of LF MF and MNPs on the spontaneous lysis of *E. coli* cells was examined at 8 × 10^9^ particles per mL and 2 × 10^7^ cells per mL, respectively. As can be seen in Figure 13, neither the LF MF and MNPs individually nor both in combination significantly affected the spontaneous lysis rate of the *E. coli* cells.

### 3.6. E. coli Cell Lysis Induced by Lys394 Recombinant Enzyme Mediated by MNPs in LF MF

**Figure 14:**
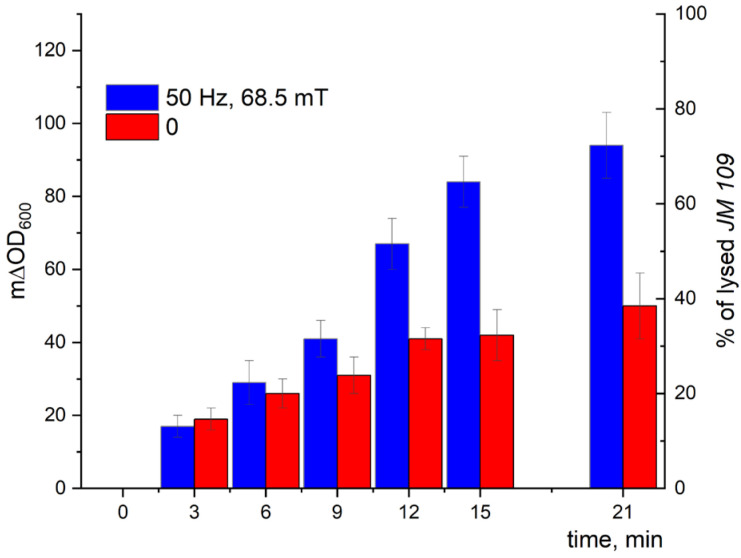
*E. coli* JM 109 cell lysis induced by Lys394 endolysin in the presence of MNPs under LF MF. The reaction was carried out in 10 mM Tris-HCl (pH 8.2) at 25 °C. MNPs, *E. coli*, and Lys394 concentrations were 8 × 10^9^ particles/mL, 2 × 10^7^ cells/mL, and 4.8 µg/mL, respectively. The left axis corresponds to the change in turbidity, and the right axis corresponds to the percentage of lysed *E. coli* cells. The 100% value corresponds to cell lysis caused by sonication.

**Figure 15:**
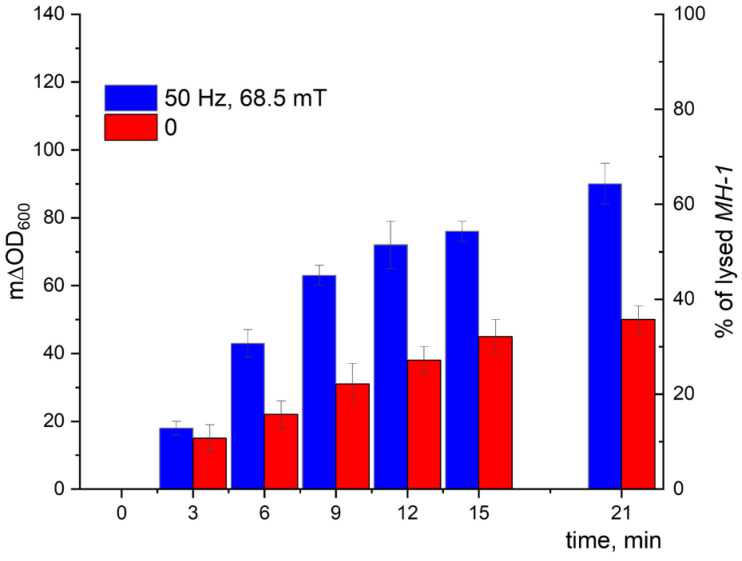
*E. coli* MH 1 cell lysis induced by Lys394 endolysin in the presence of MNPs under LF MF. The reaction was carried out in 10 mM Tris-HCl (pH 8.2) at 25 °C. MNPs, *E. coli*, and Lys394 concentrations were 8 × 10^9^ particles/mL, 2 × 10^7^ cells/mL, and 6.0 µg/mL, respectively. The left axis corresponds to the change in turbidity, and the right axis corresponds to the percentage of lysed *E. coli* cells. The 100% value corresponds to cell lysis caused by sonication.

The kinetic curves of the turbidity changes with time demonstrated a much stronger effect of LF MF mediated by MNPs on the cell lysis induced by Lys394 endolysin (blue bars) in comparison with using MNPs without LF MF (red bars), as shown in Figure 14 and Figure 15. As seen from the right axes corresponding to the percentage of lysed *E. coli* cells, almost 80 and 70% of the lysed cells appeared within 21 min of the enzyme action on the JM 109 and MH-1 strains under LF MF, respectively. There was some lysis of *E. coli* cells (less than 40%) induced by Lys394 endolysin without LF MF, as can be seen in Figure 14 and Figure 15 (red bars). This was due to the use of frozen–thawed cells, the membrane integrity of which was partially broken.

### 3.7. The Study of the Outer Membrane Permeability of E. coli 

The effect of LF MF on the outer membrane permeability in the presence on MNPs was studied for *E. coli* JM 109 cells using two independent experiments. The first one was attributed to β-lactamase and its possible leakage from the cell periplasm to the outer buffer suspension medium. To determine the amount of released enzyme, its activity was measured in the outer solution. For the second one, Nile Red hydrophobic dye was used, and its fluorescence changes were detected.

#### 3.7.1. The Detection of Periplasmic β-Lactamase Leakage

**Figure 16:**
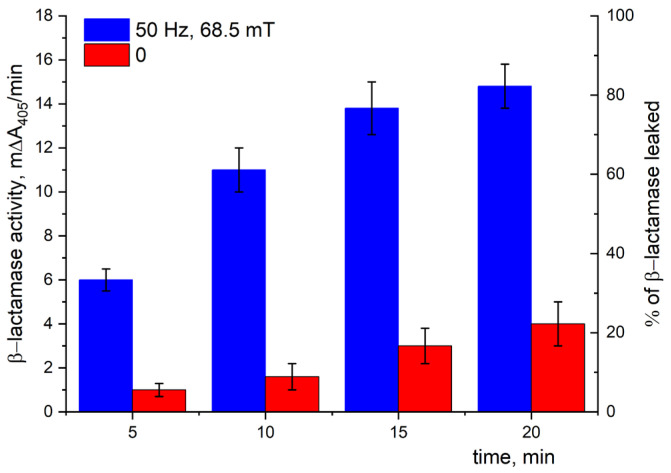
Periplasmic β-lactamase leakage in the presence of MNPs with (blue bars) or without (red bars) LF MF action. The enzyme activity was tested on JM 109 *E. coli* strain at 25 °C using chromogenic CENTA substrate. The β-lactamase activity (expressed as mΔA_405_/min) is shown on the left *Y*-axis. Each column corresponds to the percentage of β-lactamase leaked (on the right *Y*-axis), where 100% leakage corresponds to the cell disruption by sonication. All values are averages of three measurements ± standard deviation.

Figure 16 presents the time dependence of the β-lactamase activity in an outer solution (buffer suspension medium). This activity was related to the enzyme coming out of the periplasm of *E. coli* to the outer medium in the presence of MNPs exposed to LF MF. As can be seen (Figure 16, blue bars), the β-lactamase activity indeed increased over time with the action of LF MF, i.e., there was an increase in the β-lactamase concentration in the outer solution. It should be noted that the enzyme can only leave the periplasm if the membrane integrity is broken. Exposure to LF MF resulted in an increase in β-lactamase of up to more than 80% in 20 min (Figure 16, blue bars). Note, about 20% of the β-lactamase was observed in the outer solution without LF MF. The small leakage of β-lactamase in the control sample can be explained by spontaneous lysis over time and/or by partly broken cell walls due to the usage of frozen–thawed cells (Figure 16, red bars).

#### 3.7.2. Application of Hydrophobic Nile Red Dye

**Figure 17:**
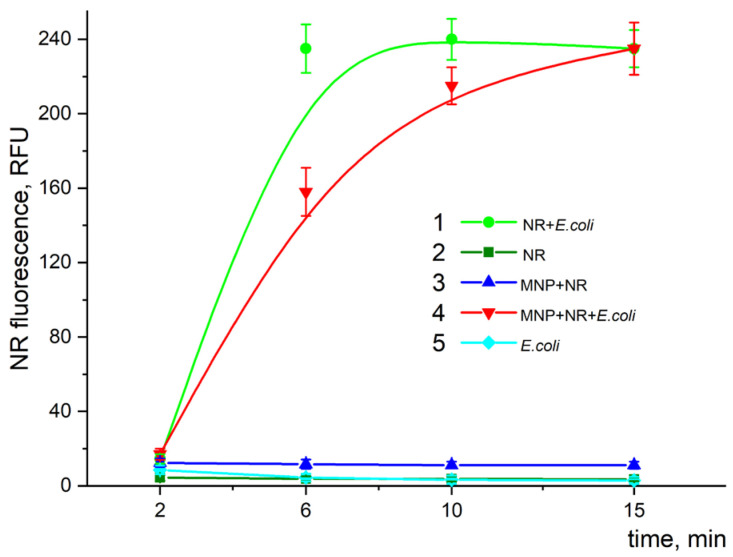
Time dependence of fluorescence intensity of NR in different reaction compositions: 1—mixture of *E. coli* with dye; 2—dye in buffer solution; 3—dye with MNPs in buffer solution; 4—dye mixture with particles and cells; 5—cells only. Cells, MNPs, and NR concentrations were 2 × 10^7^ cells/mL, 8 × 10^9^ particles/mL, and 0.4 µg/mL, respectively. The reaction was carried out in Tris-HCl (pH 8.2) buffer at 25 °C. The fluorescence intensity was measured at the maximum of the fluorescence peak λ_em_ at 612 nm (λ_ex/em_ = 545/612 nm).

Nile Red (NR) is a dye that only displays a strong fluorescence in a hydrophobic environment. The time dependence of the fluorescence intensity of NR at λ_ex/em_ = 545/612 nm is shown in Figure 17 for the individual components of the original reaction mixture and their various combinations. As can be seen, significant fluorescence was observed just in two compositions that contained bacterial cells, while in all the other cases, the fluorescence was negligible (close to the control sample without any dye) (compare cyan dots, green squares, and blue triangles in Figure 17). The fluorescence intensity in the mixture containing cells and NR (green dots) increased with time, reaching its saturation level at about 6 min. The addition of MNPs to this mixture (red triangles), as can be seen, slightly slowed down the fluorescence growth (15 min).

**Figure 18:**
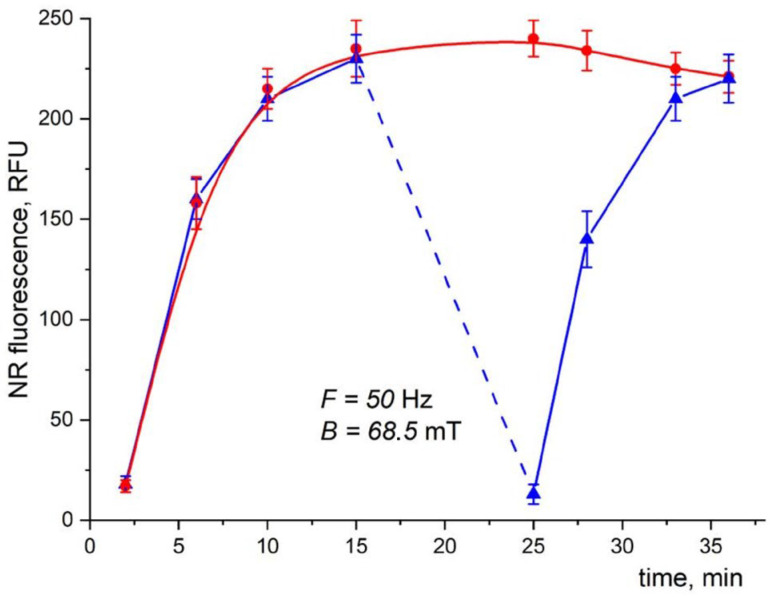
Time dependence of fluorescence intensity in the reaction mixture containing cells, MNPs, and NR dye with (blue triangles) and without (red dots) LF MF action. The dotted line indicates the time of LF MF exposure. Cells, MNPs, and NR concentrations were 2 × 10^7^ cells/mL, 8 × 10^9^ particles/mL, and 0.4 µg/mL, respectively. Reactions were conducted in 10 mM Tris-HCl buffer (pH 8.2) at 25 °C, λ_ex/em_ = 545/612 nm.

The effect of LF MF on the fluorescence intensity of NR in the mixture containing MNPs was studied (Figure 18, blue triangles). After 15 min of incubation without LF MF, when the fluorescence intensity reached its saturation level, the sample was exposed to LF MF for 5 min. As can be seen, the NR fluorescence drastically dropped down to the initial level. After the LF MF exposure stopped, the fluorescence intensity started increasing again, reaching its saturation. The reference sample (Figure 18, red spheres) without exposure to LF MF showed an increase in fluorescence and a saturation that remained almost unchanged for the whole duration of the experiment.

## 4. Discussion

The current work opens new ways of significantly increasing the lysis of *E. coli* JM 109 and MH 1 cells induced by Lys394 endolysin in the presence of MNPs exposed to a low-frequency alternating magnetic field in a pulse–pause mode (1 s on and 0.3 s off). As shown previously in [25], the pulse–pause LF MF mode gives a much greater effect than the continuous mode when used for biological objects. A low frequency and moderate intensity of the magnetic field employed ensures that neither local nor overall heating of the suspension is observed. The heat release rate was negligibly lower than that observed with magnetic hyperthermia, which employs a magnetic field with frequencies around 200–300 kHz [49]. Estimates show [49,50] that the maximum local heating that can occur in a medium with MNPs in the field of <100 mT and 50 Hz does not exceed 0.001 K, and the total heating of the suspension by the field is even less. Therefore, the nanomechanical deformation of the macromolecules is the determining factor in the exposure of systems with MNPs to LF MF.

Rod-like MNPs were synthesized and functionalized by dopamine for the current study. The choice of the morphology of the MNPs was based on the results of the theoretical calculations and experimental data presented in [25,26], according to which rod-like nanoparticles have a more significant effect on disordering biological membranes relative to spherical particles. Dopamine gives additional positive charges to and increases the stability of MNPs. In this study, both the MNPs and β-akaganeite were characterized using NTA, Mössbauer spectroscopy, and TEM. The magnetic curve of the MNPs was also obtained. As follows from the Mössbauer spectroscopy data, the first stage of the synthesis resulted in β-akaganeite nanoparticles, and the final product of the synthesis was a mixture of the following components: β-akaganeite, magnetite, and maghemite (see Figure 2 and Figure 3 and Table 1 and Table 2). The Mössbauer spectrum of MNPs consisted of three sextets and two quadruple doubles. The latter ones were the main contribution to the spectrum (see Figure 3). The sextets that emerged in the spectrum provided evidence for the formation of the magnetic phases of the magnetite and maghemite. The spectrum approximation yielded the following composition of the MNPs: 74% β-akaganeite, 19% magnetite, and 7% maghemite. The average dimensions of the β-akaganeite precursor particles and MNPs differed only slightly. However, a reduction in the amount of β-akaganeite led to a narrowing of the length distribution of the MNPs; furthermore, the formation of larger particles was accompanied by a diminishing in the number of smaller ones (Figure 5 and Figure 7). This might be the result of a recrystallization process [36] that led to the particles coarsening. The increase in the average hydrodynamic radius of the particles from 77 nm to 84 nm and the decrease in their concentration from 6 × 10^15^ to 2 × 10^15^ particles per mL supports the above considerations. The magnetization curve of the MNPs, i.e., the dependence of its magnetic moment on the magnetic field intensity, is shown in Figure 9. The MNPs used in this work presented ferrimagnetic properties, and their coercive force (H_c_) of 9.6 kA/m as presumably a result of the anisometric shape of the MNPs.

We used the *E. coli* JM 109 and MH 1 cell strains as a substrate and Lys394 endolysin as a bacteriolytic enzyme in this study. The activity of the Lys394 endolysin with these substrates is shown in Figure 11. As can be seen, the curve of the dependence of the cell lysis rate on the enzyme concentration was a complex one (nonlinear). For further experiments, we chose the enzyme concentration in the linear region of the curve. The reasoning behind this choice was based on the dominating mechanism of the lysis, which was due to peptidoglycan hydrolysis in the linear range and cell rupture osmosis in the nonlinear one. To study the effect of LF MF on the cell lysis, we chose the same initial reaction rate for the JM 109 and MH 1 cell lines with close enzyme concentrations. The concentration of MNPs chosen for the LF MF activation experiment was based upon the results of the cell lysis test conducted without LF MF. As can be seen in Figure 12, the dependence of the activity of the Lys394 endolysin on the MNP concentration in the range from 10^7^ to 10^15^ particles per mL had two distinctive parts. It was nearly constant at MNP concentrations below 10^11^ particles per mL and rapidly diminished above that value for both cell lines. The decrease could possibly be a consequence of the membrane being shielded by the large number of MNPs adsorbed on the membrane that prevents the penetration of the enzyme molecules to peptidoglycan.

To evaluate the effect of LF MF on the cell lysis, it was important to use MNPs in concentrations that did not significantly affect the spontaneous cell lysis rate. Thus, the MNP concentration of 8 × 10^9^ particles per mL was chosen for further experiments. A set of *E. coli* spontaneous lysis reference tests, including exposing the mixtures with and without MNPs to LF MF, were carried out. As can be seen in Figure 13, there was no noticeable spontaneous lysis of the *E. coli* cells with 20 min of incubation without the enzyme, and the presence of MNPs did not result in any change.

Using the above-mentioned MNPs and Lys394 endolysin concentrations, the effect of LF MF on the cell lysis was studied. As shown in Figure 14, the effect of the LF MF mediated by MNPs produced an almost twofold acceleration in the enzyme reaction rate, giving values of almost 80 and 70%, respectively, of lysed *E. coli* JM 109 and MH 1 cells in 21 min. One of the reasons for the observed effect is the disordering of the cell outer membrane caused by the rotatory oscillation of the MNPs absorbed on the cell wall that facilitated enzyme penetration through the outer membrane to reach the peptidoglycans in periplasm.

To confirm the above assumption concerning the effect of LF MF, two additional independent experiments on JM 109 cells were carried out. The first one was aimed at studying the change in the permeability of the outer membrane of *E. coli* by examining β-lactamase leakage from the periplasm. The second one was aimed at studying the possible changes in the fluorescence of a hydrophobic dye, which can be considered as a qualitative estimation of the disordering of the cell membrane. Indeed, by measuring the enzyme activity in the outer buffer solution, we confirmed that β-lactamase was released from the cell periplasm. It was shown that the disordering of the outer membrane of the *E. coli* caused by the movement of nanoparticles exposed to LF MF led to an almost complete (more than 80%) β-lactamase release out of the cells’ periplasm to the buffer suspension, revealing fourfold increase in β-lactamase activity in the outer medium compared to the reference one without LF MF action (see Figure 16). Thus, one can see that the complex oscillations of MNPs in LF MF really affected the membrane integrity, increasing its permeability to the enzyme molecules. In the second independent experiment, we employed Nile Red (NR), hydrophobic fluorescent dye. It is one of the most well-known fluorescent dyes with prominent solvatofluorochrome properties and is widely used in studying the fluorescence of biological objects [41], which is quite sensitive to environmental changes and is sharply increased in non-polar media [41]. In our case, the non-polar environment was provided by the hydrophobic region of the outer membrane of *E. coli*. Figure 17 presents the time dependence of the fluorescence intensity of NR for the various components in the reaction mixture. It should be noted that the cells and NR in the buffer solution individually exhibited a very low level of fluorescence regardless of the presence of MNPs. Considering that NR fluorescence is only observed in hydrophobic environment, in our case, it was only seen in the mixtures containing bacterial cells. The difference in the time in which the fluorescence reached its saturation level was presumably related to the shielding effect of the MNPs absorbed on the cell wall that hindered the permeation of the NR into the hydrophobic region of the lipid bilayer. The data shown in Figure 18 revealed an order of magnitude drastic reduction in the fluorescence intensity of the NR, reaching the initial extremely low level for the reaction mixture containing MNPs, cells, and NR when exposed to LF MF. The switching off of the exposure to LF MF resulted in the fluorescence of the NR increasing back to the saturation level. Thus, the effect was reversible, meaning that the cell membrane was disordered, but the cell wall was not ruptured. Two models could be proposed to explain the effect observed. The first one implies that the NR molecules were expelled from the membrane into the buffer suspension when the cell membrane lost its integrity. The second one relies on the partial hydration of hydrophobic region of the outer membrane due to water molecules penetrating into the disordered cell membrane that could affect the environmental polarity of the NR molecules and, hence, their fluorescence intensity.

## 5. Conclusions

The current paper reported a study on boosting the lysis of *E. coli* JM 109 and MH 1 cells induced by Lys394 bacteriophage endolysin in the presence of rod-like magnetic nanoparticles activated by a non-heating low-frequency alternating magnetic field in a pulse–pause mode. It was found that exposure to LF MF resulted in a significant acceleration of the enzyme activity compared to the reference one. We revealed that the acceleration of the cell lysis was associated with a disordering of the cell outer membrane caused by the complex oscillating motion of the MNPs in LF MF. The data obtained provide evidence for structural changes in the bacterial cell wall. This effect was confirmed by two additional independent experiments showing the release of β-lactamase out of the periplasm into an outer medium and a significant change in the fluorescence of Nile Red.

Thus, the possibility of remotely controlling the enzymatic lysis of bacterial cells based on the disordering of their outer membranes with rod-like MNPs in an external non-heating low-frequency alternating magnetic field that causes the oscillatory motion of the MNPs is revealed.

This study demonstrates and opens up the prospect of the application of an external non-heating low-frequency alternating magnetic field for the remote controlling of bacteriophage endolysin antimicrobial activity and its enhancement against Gram-negative pathogens.

## Figures and Tables

**Table 1:** Hyperfine parameters obtained from the fit of the experimental Mössbauer spectra of the precursor. Isomeric shifts are given relative to α-Fe at RT.

Peak No.	Is, mm/s	Qs, mm/s	S Ref., %
D-1	0.37	0.56	68
D-2	0.36	0.99	32

Estimated errors of Is, Qs, and S were: ±0.05 mms/s, ±0.05 mms/s, and ±5%, respectively.

**Table 2:** Hyperfine parameters obtained from the fit of the experimental Mössbauer spectra of the final nanorods. Isomeric shifts are given relative to α-Fe at RT.

Peak No.	Is, mm/s	Qs, mm/s	H, kOe	S Ref., %	G, mm/s
S-1 S-2 S-3 D-1	0.22 0.58 0.33 0.37	0.30 0.11 −0.02 0.60	482 450 495	6 13 7 55	0.77 0.78 0.56 0.38
D-2	0.36	1.00		19	0.39

Estimated errors of Is, Qs, H, S, and G were: ±0.05 mms/s, ±0.05 mms/s, ±5 kOe, ±5%, and ±0.03 mms/s, respectively.

## Data Availability

Not applicable.

## Abbreviations

The following abbreviations are used in this manuscript:

LF MF	Low-frequency magnetic field
MNPs	Magnetic nanoparticles
NR	Nile Red
NTA	Nanoparticle tracking analysis
TEM	Transmission electron microscopy
*E. coli*	*Escherichia coli*
RT	Room temperature
